# Effect of sous vide cooking combined with bromelain on beef tenderness and eating quality

**DOI:** 10.1016/j.fochx.2025.103438

**Published:** 2025-12-22

**Authors:** Wei Huang, Lijing Geng, Hang Fu, Dan Wang, Dongsheng Jia, Ao Wang, Rong Qi, Mbinga Isequias Elamba Tertulliano, Muhammad Zain Ul Aabideen, Wei Zhou, Quimbamba Silvia Jacinta Calombe, Aqsa Shafique, Chang Liu

**Affiliations:** a College of Food and health, Jinzhou Medical University, Jinzhou 121001, China; b Key Laboratory of Molecular Cell Biology and New Drug, Jinzhou Medical University, Jinzhou 121001, China; c Liaoning Province meat processing and quality safety control professional technology innovation center, Jinzhou 121001, China; d College of Clinical Medicine, Jinzhou Medical University, Jinzhou 121001, China; e College of Pharmacy, Jinzhou Medical University, Jinzhou 121001, China

**Keywords:** Sous vide cooking, Bromelain, Beef, Principal component analysis, Eating quality

## Abstract

To enhance beef tenderness and eating quality, this study optimized the sous vide (SV) cooking process combined with bromelain treatment using response surface methodology. Conventional cooking served as the control, while SV cooking and SV cooking combined with bromelain treatment (SV + Bro) comprised the experimental groups. Beef quality was evaluated by measuring shear force, cooking loss, texture profile, color difference. Results showed optimal conditions included 250 U/mL bromelain, an SV cooking temperature of 65 °C, and an SV cooking time of 120 min. Compared to the control, SV + Bro reduced shear force by 37.710 %, cooking loss by 12.342 %, and increased immobilized water and the myofibril fragmentation index. Principal component analysis further revealed the SV + Bro group had the highest comprehensive score. This study validated the synergistic effect of SV and bromelain in improving beef eating quality, providing theoretical support for its application in meat processing.

## Highlights

• SV cooking was combined with bromelain for the first time.
• RSM optimized SV cooking combined with bromelain tenderizes beef.
• Microscopic structure reveals higher fragmentation in synergistic myofibril processing.
• SV cooking combined with bromelain was a better cooking method.

## Graphical abstract

**Figure:**
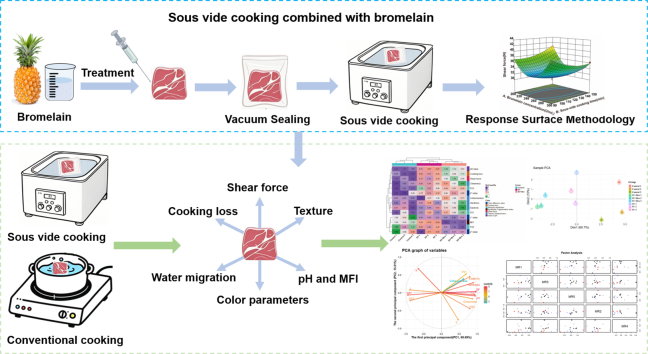

## 1 Introduction

The eating quality of meat was primarily determined by tenderness and juiciness. Tenderness, as a critical sensory attribute of cooked meat, directly affected the purchasing intent of consumers (Polkinghorne & Thompson, 2010). Research indicated that if beef possessed good tenderness and higher overall edible quality, consumers were generally willing to pay a higher price for it (N'gatta et al., 2022). However, providing consumers with high-quality texture and an excellent eating experience in beef had always been a significant challenge faced by the beef industry. Therefore, tenderization treatment during beef processing was particularly important.

Currently, meat tenderization techniques include high-pressure processing, ultrasonic treatment, enzymatic processing, electrical stimulation, and sous vide (SV) cooking, etc. (Bhat et al., 2018). Among these, SV cooking was a thermal processing technique in which meat was placed in vacuum-sealed bags and heated in a water bath under precisely controlled temperature and time conditions, with heating temperatures typically ranging between 50 and 85 °C (Latoch et al., 2023). Compared to conventional cooking techniques, SV cooking offered several advantages, such as reducing cooking losses (Silva et al., 2016), preserving nutritional substances (Chew et al., 2024), extending shelf life (Bıyıklı et al., 2020), reducing lipid and protein oxidation (Ortuño et al., 2021), enhancing sensory characteristics (Bıyıklı et al., 2020), and increasing meat tenderness (Chen et al., 2025). Research by Djekic et al. (2021) indicated that chicken exhibited the lowest hardness and best tenderness compared to other cooking methods. Jeong et al. (2018) also found that SV cooking improved tenderness of pork ham compared to other methods. However, to achieve the desired tenderness for consumers, SV cooking required prolonged heat treatment, which not only led to lipid oxidation and undesirable odors but also affected the appearance of the meat and increased energy consumption (Oliveira et al., 2024). Therefore, there was an urgent need to find a solution that could leverage the advantages of SV cooking while effectively overcoming the drawbacks of extended heat treatment.

Plant proteases, due to their characteristics of being naturally pollution-free, widely sourced, and possessing high hydrolytic activity, were widely applied in meat tenderization (Mohd Azmi et al., 2023). Bromelain, a natural plant protease extracted from pineapple, was classified as a cysteine protease (Bekhit et al., 2014). It acted on myofibrillar proteins and connective tissues, hydrolyzing them into small molecular peptides and amino acids, thereby improving the tenderness and reducing the hardness of the meat within certain limits (Gallego et al., 2023). Therefore, this study combined SV cooking with bromelain to explore their synergistic tenderization effect, addressing the limitations of using SV cooking alone. The optimal conditions for the combined treatment were determined using response surface methodology (RSM). Additionally, the impact of various cooking techniques on the quality of beef was examined utilizing cluster analysis and principal component analysis (PCA). This study not only provided a scientifically effective method for improving beef tenderness but also established a theoretical basis for its use in meat processing.

## 2 Materials and methods

### 2.1 Materials and reagents

Longissimus thoracis (LT) muscles from three 3-year-old Simmental cattle, obtained 48 h post-mortem, were purchased from Huazi Beef and Mutton Wholesale and Retail Store in Guta District, Jinzhou, Liaoning Province; the cattle had a carcass weight of 200 ± 30 kg (three pieces totaling 20 kg). After purchase, the meat was immediately packed into insulated containers with ice packs and transported to the laboratory (approximately 2 °C). The item was stored overnight in the refrigerator at 4 °C. Bromelain (food grade, 100,000 U/g) was purchased from Nanjing Do-Li Biotechnology Co., Ltd. (Nanjing, China). Disodium phosphate, sodium dihydrogen phosphate, magnesium chloride, EDTA, and sodium chloride (all of analytical grade) were obtained from Tianjin Fengchuan Chemical Reagent Technology Co., Ltd. (Tianjin, China).

### 2.2 Beef tenderization processing by SV cooking combined with bromelain

Before the experiment commenced, visible fascia was removed, and each muscle was divided into 40 pieces, each measuring 4 × 4 × 5 cm^3^ and weighing 100 ± 5 g. These pieces were then randomly assigned to various experimental groups. The treatment method of bromelain was slightly modified based on the approach described by Ma et al. (2019). A stock solution with a concentration of 1000 U/mL was prepared by dissolving 1 g of bromelain (100,000 U/g) into 100 mL of 0.1 mol/L phosphate-buffered saline (PBS, pH = 7.0). The stock solution was then diluted with PBS to prepare enzyme solutions with final concentrations of 100–300 U/mL (0.10–0.30 %, *w*/*v*). Subsequently, 10 mL of the enzyme solution was uniformly injected into 100 ± 5 g of meat along the direction of muscle fibers. After injection, the meat was hand-massaged for 1 min to ensure even distribution of the solution. The treated meat piece was then sealed in a food-grade vacuum packaging bag. The sample was incubated in a water bath (Jinghong Laboratory Instrument Co., Shanghai, China) at 30 °C for 30 min and then immediately transferred to a constant temperature water bath set at the target temperature. The heating time was measured from the point when the core temperature of the meat reached the set value. Throughout the process, acalibrated needle-type thermocouple was used to measure the core temperature. Upon completion of heating, the sample was removed and quickly immersed in an ice-water bath to terminate the process, reducing the impact of residual heat on beef quality. Subsequently, relevant indicators were quickly measured.

For the single-factor analysis, the bromelain concentration treatment (ranging from 100 to 300 U/mL) was applied with an SV cooking time of 120 min and an SV cooking temperature of 65 °C. The SV cooking time treatment (ranging from 60 to 180 min) was conducted with 250 U/mL bromelain concentration and an SV cooking temperature of 65 °C. The SV cooking temperature treatment (ranging from 55 to 75 °C) was applied with 250 U/mL bromelain concentration and an SV cooking time of 120 min. The tenderization of beef was assessed by measuring shear force. Each treatment was performed in triplicate.

Response surface methodology (RSM) effectively assessed the relationships and interactions among the three independent factors. Following the single-factor tests under the specified conditions, the combined tenderization treatment was further refined using RSM with Design Expert software (version 8.0.6). For experimental design, the following variations were employed: bromelain concentration (200 to 300 U/mL), SV cooking time (90 to 150 min), and SV cooking temperature (60 to 70 °C) (Table S1). The design included 17 experimental runs, detailed in Table S2. A second-order regression model was used to evaluate the correlation among these factors:

*Y* = β_0_+ β_A_ *A* + β_B_ *B* + β_C_ *C* + β_AB_ *AB* + β_AC_ *AC* + β_BC_ *BC* + β_AA_ *A*^2^ + β_BB_ *B*^2^ + β_CC_ *C*^2^ + ε.

where β_0_ represented the constant term, β_A_, β_B_, and β_C_ were the coefficients of the linear terms, β_AB_, β_AC_, and β_BC_ were the coefficients of the interaction terms, β_AA_, β_BB_, and β_CC_ were the coefficients of the quadratic terms, and ε was the random error.

The optimal combined tenderization treatment for beef, as determined by RSM, was designated as the SV + Bro group. The group without bromelain, subjected only to SV cooking (65 °C, 120 min), was labeled as the SV group. The control group underwent traditional boiling for 120 min without bromelain or vacuum packaging.

### 2.3 Shear force

Beef samples were sliced into strips measuring 1 × 1 × 3 cm^3^ along the grain and positioned on the cutting platform of a TA-XT texture analyzer (Stable Micro System Inc., UK), using a TA/LKB light cutter to cut perpendicularly to the muscle fibers. The test parameters were set to single test mode with a deformation target of 99 %. The pre-test, mid-test, and post-test speeds were set at 2, 1, and 2 mm/s respectively, and measurements were performed in parallel five times.

### 2.4 Cooking loss

The initial weight of the sample was recorded as m_1_ before heating, and after heating, the surface moisture of the sample was blotted and weighed, noted as m_2_. The formula for calculating the cooking loss was as follows (1):

(1) $$\mathrm{Cooking loss}\;\left(\%\right)=\frac{m_{1}-m_{2}}{m_{1}}$$

### 2.5 Texture profile analysis

Following the method by Wang et al. (2016) with minor adjustments, the texture analysis was carried out. The processed samples were cut into 1 × 1 × 1 cm^3^ cubes and placed on the TA-XT texture measurement platform. A P/36R probe was used for the two-cycle compression mode, pressing vertically along the direction of myofibrils, with an interval of 3 s between the two compressions. The parameters were set for a full texture test, with the target mode as deformation, and a target value of 30 %. The pre-test, mid-test, and post-test speeds were all set at 2 mm/s, with a trigger force of 5 g. Measurements were conducted in parallel five times. Hardness, elasticity, chewiness, and cohesiveness were measured.

### 2.6 Color parameters

Following the slicing of the cooled samples, they underwent a blooming process at temperature (6–7 °C) for 30 min. Subsequently, the beef's color difference parameters were assessed using a CR-400 colorimeter (Konica Minolta, Japan). The instrument was calibrated with a standard white reference plate, and the illuminant was configured to CIE D65, while the standard observer angle was established at 2 degrees. The measurement aperture was set to 8 mm. The probe was positioned vertically against the sliced surface of the beef to gauge the values of lightness (L*), redness (a*), and yellowness (b*). Each sample was subjected to three measurements, and the average values were computed.

### 2.7 pH value

A 5 g sample was weighed and added to 25 mL of distilled water. It was homogenized at a speed of 8000 r/min for 1 min using a homogenizer (XHF-DY, Xinzhi Biotechnology Co., Ningbo, China). The pH value of the sample was then measured with a pH meter, which had been calibrated at the present environmental temperature with pH buffer solutions of 4.01 and 6.86. The pH measurement was repeated three times, and the average value was recorded.

### 2.8 Myofibril fragmentation index (MFI)

The determination of MFI was conducted with slight modifications to the method proposed by (Soltanizadeh et al., 2008). Beef was minced, with 4 g weighed and homogenized for 30 s in MFI buffer at 10 times the volume (0.1 mol/L NaCl, 20 mmol/L Na_2_HPO_4_/NaH_2_PO_4_, 2 mmol/L MgCl_2_, 1 mmol/L EGTA; pH = 7). It was then centrifuged at 8000 r/min for 10 min, after which the supernatant was removed. This process was carried out three times. The resulting myofibril pellets were resuspended in MFI buffer at 4 °C, and the protein concentration was finally modified to 0.5 mg/mL. Lastly, absorbance at a wavelength of 540 nm was recorded using a UV/Vis spectrophotometer (UV-2802; UNICO Instruments Co., Shanghai, China). The MFI was calculated using the following Eq. (2):

(2) $$\mathrm{MFI}=200\times {\mathrm{Absorbance}}_{540\;\mathrm{nm}}$$

### 2.9 Low field nuclear magnetic resonance (LF-NMR)

The methodology based on Cao et al. (2018) was modified slightly to prepare the samples by cutting them into small blocks measuring 0.5 × 0.5 × 2 cm^3^, which were then placed in Nuclear Magnetic Resonance (NMR) tubes for LF-NMR analysis. The parameters were configured as follows: a proton resonance frequency of 20 MHz, a sampling frequency of 250 kHz, a repetition time (TR) of 3000 ms, and an echo time (TE) of 20 ms. After the detection process was completed, data collection took place, followed by the execution of a T_2_ inversion program to analyze the distribution of relaxation times.

To assess the proton density spectra, Magnetic Resonance Imaging (MRI) was employed with the following parameters: TR was 500 ms, and TE was 18.2 ms. The imaging layer was chosen according to the Larmor relation, along with adjustments made for both the signal-to-noise ratio and the clarity of the images. Each sample was subjected to four repeated samplings, and the experiment was conducted three times.

### 2.10 Clustering and principal component analysis (PCA)

In order to investigate the effects and interactions of shear force, cooking loss, texture, color parameters, pH value, MFI, and moisture distribution peak area ratio on beef quality under different cooking methods, a study was conducted using cluster and PCA. PCA was applied to extract key factors through dimensionality reduction, and to construct principal component score equations that integrated multiple variables. A detailed assessment framework was created to examine how different cooking techniques affect the quality of beef.

### 2.11 Histological analysis

The histological changes in beef were observed using hematoxylin and eosin (H&E) staining. The procedure was based on the method described by Xu et al. (2020) with slight modifications. Initially, the samples were fixed with 3 % glutaraldehyde. Following fixation, samples underwent dehydration using an ethanol solution. After dehydration, the muscle tissue was embedded in paraffin and then sectioned into 5 μm thick slices using a microtome. Finally, the tissue sections were stained with H&E, and histological observations were conducted under an optical microscope (model CX23, Olympus Co., Ltd., Beijing, China), with simultaneous recording of the microstructural changes.

### 2.12 Statistical analysis

The data were expressed as mean ± standard deviation, with each experiment repeated at least three times. Statistical analysis was conducted using linear mixed model (LMM) constructed with SPSS version 27 software (IBM Corp, USA). The amount of bromelain addition, SV cooking time, and SV cooking temperature were set as fixed effects, including the interactions among the amount of bromelain addition, SV cooking time, and SV cooking temperature. Meanwhile, carcasses and experimental batches were treated as random effects to control for their influence on result variance. The significance of fixed effects on various indicators was assessed by analysis of variance (ANOVA) within the model. Duncan's multiple range test was used to compare the significance of differences between groups, with *P* ≤ 0.05 indicating significance. A Box-Behnken response surface design was constructed via Design-Expert 13 software (Stat-Ease Inc., USA), using a quadratic polynomial regression model to analyze main effects, secondary effects, and interaction effects, and to optimize process parameters. Before constructing the response surface model, the data were corrected using the LMM results. Data visualization was carried out using Origin 2021 software (Origin Lab Corp, USA). PCA was performed on multi-index data using R software, and comprehensive principal components were extracted using the Varimax rotation method to evaluate the overall quality differences among groups. All statistical models were refined using the stepwise backward elimination method to exclude non-significant variables. *P* ≤ 0.05 was considered statistically significant.

## 3 Results and discussion

### 3.1 Single-factor experiment

**Fig. 1:**
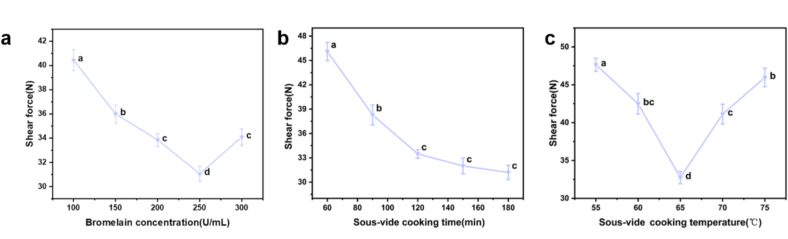
Single-factor trial results. (a) Bromelain concentration. (b) SV cooking time. (c) SV cooking temperature. The results were presented as mean ± standard deviation, with different letters indicating significant differences among the samples (*P* < 0.05).

As shown in Fig. 1a, the shear force reached a minimum of 33.321 ± 0.363 N when the bromelain concentration was at 250 U/mL. Further increases in bromelain concentration resulted in a rise in shear force, possibly attributable to excessive enzymatic proteolysis by bromelain, leading to the degradation of beef muscle fibers, which in turn caused dehydration, making the meat tougher (Abdel-Naeem & Mohamed, 2016). The research conducted by Sun et al. (2023) aligns with the results of this study. When papain with a concentration gradient of 40, 80, 120, 160, and 200 U/mL was used to tenderize camel meat. It was found that at the papain concentration of 120 U/mL, the shear force of camel meat was the lowest. Concurrently, the water-holding capacity (WHC) reached its maximum value of (73.04 ± 1.31 %). in contrast, when the enzyme concentration was increased to 200 U/mL, the shear force of camel meat increased significantly, while the WHC decreased to 67.45 ± 0.46 %. As illustrated in Fig. 1b, the shear force of beef significantly decreased with extended SV cooking time, with no significant difference after a cooking time of 120 min, indicating that even a shorter cooking time achieved an acceptable level of beef tenderness. As depicted in Fig. 1c, the SV cooking temperature significantly affected the tenderization of beef (*P* < 0.05). Within the range of 55–65 °C, the shear force of beef gradually decreased and reached the minimum value of 32.743 ± 0.812 N at 65 °C. This trend was likely caused by the combined effects of bromelain treatment and sous-vide heating. Bromelain exhibited high proteolytic activity at moderate temperatures (approximately 50–60 °C) and partially degraded myofibrillar proteins during the initial phase of heating, whereas its activity was rapidly lost at higher temperatures (Bekhit et al., 2014). As the meat temperature further increased and was maintained at 60–65 °C, the thermal solubilization of intramuscular collagen was enhanced, which also contributed to improved tenderness. However, when the temperature exceeded 65 °C, thermal denaturation and contraction of myofibrillar proteins and collagen occurred, resulting in reduced water-holding capacity and increased hardness of the meat (Alahakoon et al., 2018). Therefore, the lowest shear force observed at 65 °C in our study was most likely the result of the synergistic action of prior enzymatic weakening and collagen solubilization during SV cooking. According to the findings from individual factor experiments, the ideal conditions for beef tenderization were: bromelain concentration of 250 U/mL, SV cooking time of 120 min, and SV cooking temperature of 65 °C.

### 3.2 Response surface optimization

This research involved conducting a response surface experiment that examined three factors, each at three levels. The independent variables included the quantity of bromelain concentration (A), SV cooking time (B), and SV cooking temperature (C), with shear force serving as the dependent variable. Table S2 illustrates the experimental design, and Table 1 displays the results of the variance analysis for shear force. The data from Table S2 were subjected to a fitting analysis using software, which resulted in the following quadratic regression equation: *Y* = 31.100 – 0.759 *A* + 0.099 *B* – 0.613 *C* – 1.640 *AB* – 1.240 *AC* + 0.650 *BC* + 5.790 *A*^2^ + 1.380 *B*^2^ + 4.160 *C*^2^. As illustrated in Table 1, the regression model *P* < 0.0001, while the lack of fit term *P* > 0.05, indicating that the model fit was excellent. Additionally, R^2^ of 98.810 %, and R^2^_adj_ of 97.280 % respectively, suggesting that the fitted regression equation is highly reliable. Based on the *F-*values, it was determined that the influence of each factor on the shear force of beef followed the order A > C > B. The main effects of A, B, and the quadratic effects A^2^, B^2^, C^2^, as well as the interaction effects AB and AC, were found to have significant impacts on the shear force of the beef (*P* < 0.05), while the remaining terms were not significant (*P* > 0.05).

**Table 1:** ANOVA analysis of the quadratic regression equation for shear force.

Source	Sum of Squares	DF	Mean Square	*F*-Value	*P*-value	significant
Model	267.48	9	267.48	64.58	< 0.0001	***
A	4.61	1	4.61	10.02	0.0158	*
B	0.0788	1	0.0788	0.1713	0.6914	–
C	3.01	1	3.01	6.54	0.0377	*
AB	10.81	1	10.81	23.49	0.0019	**
AC	6.20	1	6.20	13.47	0.0080	**
BC	1.69	1	1.69	3.68	0.0966	–
A^2^	141.05	1	141.05	306.51	< 0.0001	***
B^2^	7.99	1	7.99	17.36	0.0042	**
C^2^	72.95	1	72.95	158.52	< 0.0001	***
Residual	3.22	7	0.4608			–
Lack of Fit	1.38	3	0.4602	1	0.4781	–
Pure Error	1.84	4	0.4597			
Cor Total	270.70	16				
R^2^	0.9881	R^2^_adj_	0.9728			

Note: * Indicates a significant effect (*P* < 0.05), ** indicates a highly significant effect (*P* < 0.01), *** indicates an extremely significant effect (*P* < 0.0001), and – indicates an insignificant effect (*P* > 0.05). Model factors: A = Bromelain concentration (U/mL), B = SV cooking time (min), C = SV cooking temperature (°C). DF: degree of freedom.

**Fig. 2:**
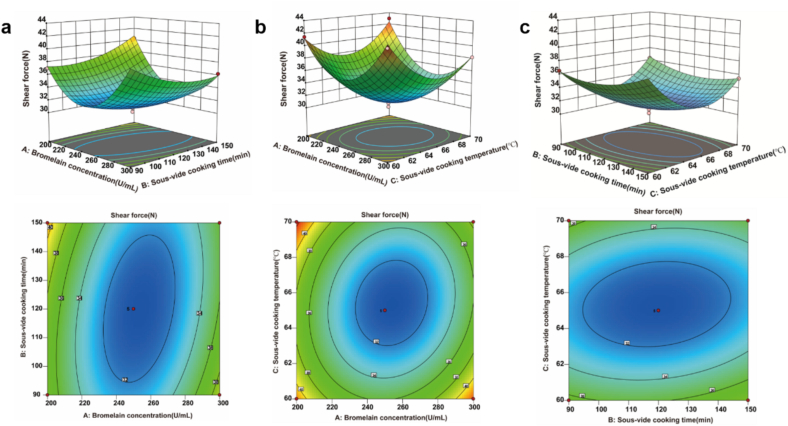
Response surface diagram of beef tenderized by SV cooking combined with bromelain. (a) Effect of bromelain concentration and SV cooking time on shear force. (b) Effect of bromelain concentration and SV cooking temperature. (c) Effect of SV cooking time and SV cooking temperature on shear force.

The effects of the interaction among A, B, and C on beef shear force are illustrated in Fig. 2. It was observed that the slopes of the response surfaces for AB and AC were steep, with densely packed elliptical contour lines, as indicated by significant differences in their *P* values (*P* < 0.05) from Table 1. This suggests that the interactions between AB and AC had a significant impact on shear force. In contrast, the response surface slopes for BC were relatively gentle, and the *P* value was not significant, indicating that the interaction between BC had no significant impact on the shear force.

The optimal processing conditions for combining SV cooking with bromelain to tenderize beef were derived using software analysis. These conditions were determined to be bromelain concentration level of 254 U/mL, SV cooking temperature of 65.438 °C, and SV cooking time of 120.969 min. Under these conditions, the predicted shear force value was 31.045 N. Considering practical feasibility, the tenderization process parameters were adjusted to bromelain concentration level of 250 U/mL, SV cooking temperature of 65 °C, and SV cooking time of 120 min. Using these optimized conditions, three parallel validation experiments were conducted, yielding an actual shear force value of 30.723 ± 0.973 N, which did not significant difference from the predicted value. Therefore, the optimization of SV combined with bromelain for beef tenderization using the response surface methodology proved to be both reasonable and reliable.

### 3.3 The effect of cooking methods on the shear force

**Fig. 3:**
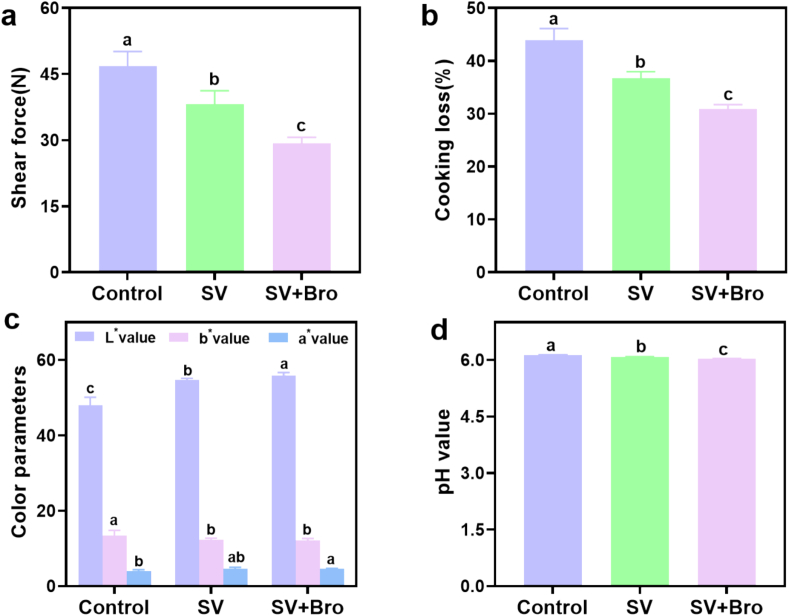
The effects of cooking methods on the shear force (a), cooking loss (b), color parameters (c), and pH value (d) of beef. The results were presented as mean ± standard deviation, with different letters indicating significant differences among the samples (*P* < 0.05).

Shear force was considered a key indicator for measuring the tenderness of meat products, the lower the shear force, the higher the tenderness (Zhang et al., 2020). As depicted in Fig. 3a, the shear force value of beef in the SV + Bro group experienced a significant reduction when compared to the control group, with a decrease of 37.710 % (*P* < 0.05). This may have been associated with the progressive thermal denaturation of muscle structure during SV cooking and the hydrolysis of myofibrillar proteins by exogenous bromelain. The results of this study align with the research conducted by Naqvi et al. (2021), who found that ginger protease added to aged cows during SV cooking notably reduced both shear force and cooking loss.

### 3.4 The effect of cooking methods on the cooking loss

The relationship between cooking loss and the juiciness as well as moisture content of the meat was significant. Fig. 3b illustrated that the cooking loss observed in the SV group and the SV + Bro group were markedly less than that of the control group (*P* < 0.05), with the SV + Bro group reduced by 12.342 % compared to the control group. This was possibly caused by the hydrolysis of muscle proteins by bromelain, which moderately disrupted the myofibrillar structure and reduced the heat-induced contraction of actomyosin. Additionally, SV cooking allowed precise control of temperature and time, maximizing moisture retention in the muscle and improving juiciness and water-holding capacity (Purslow et al., 2016). Comparable results were noted by Rasinska et al. (2019), indicating that rabbit meat exhibited reduced cooking loss when prepared using SV cooking at 72 °C for 2.5 h, in contrast to roasting at 180 °C for 60 min and boiling at 100 °C for 20 min. In a similar vein, Chang et al. (2023) found that duck breast meat showed analogous outcomes when subjected to SV cooking in comparison to pan-frying and roasting methods.

### 3.5 Texture profile analysis

Hardness is perceived through the oral experience of food's softness or firmness and corresponds to the amount of force needed to reach a specific level of deformation. Chewiness is the result of the combined effects of hardness, elasticity, and cohesiveness (Li et al., 2020). According to Table 2, the SV and SV + Bro groups exhibited decreased levels of hardness, elasticity, chewiness, and cohesiveness compared to the control group. Nonetheless, the differences noted in elasticity and cohesiveness between the SV group and the control group were not statistically significant (*P* > 0.05). This was mainly due to the mild heating conditions, which caused less heat-induced shrinkage and denaturation of the myofibrillar proteins in the meat, thereby reducing the cross-linking between macromolecules. Furthermore, the mild heating conditions of SV cooking facilitated the enzymatic activity of bromelain, resulting in a synergistic effect that significantly reduced the hardness, elasticity, chewiness, and cohesiveness of the beef. Zhao et al. (2024) found that when SV cooking marination was compared to traditional marination of duck legs, the hardness, elasticity, chewiness, and cohesiveness were reduced by 43.20 %, 29.52 %, 65.08 %, and 20.23 %, respectively, but remained within acceptable ranges, similar to the results of this study.

**Table 2:** The influence of cooking methods on the texture of beef.

Group	Hardness (N)	Elasticity (mm)	Chewiness (N)	Cohesiveness
Control	21.150 ± 4.417^a^	0.606 ± 0.069^a^	6.974 ± 1.266^a^	0.667 ± 0.014^a^
SV	12.881 ± 3.406^b^	0.579 ± 0.024^a^	4.073 ± 1.448^b^	0.644 ± 0.043^a^
SV + Bro	4.983 ± 1.071^c^	0.409 ± 0.065^b^	2.794 ± 1.404^b^	0.581 ± 0.042^b^

Note: The results were presented as mean ± standard deviation, with different letters indicating significant differences among the samples (*P* < 0.05).

### 3.6 Color parameters analysis

From Fig. 3c, the L* value for the SV + Bro group was significantly increased relative to that of the control group (*P* < 0.05). This may be partly attributed to the slower heat transfer rate and prolonged heating time during SV cooking, and partly due to light scattering. In this study, the SV + Bro group exhibited lower cooking loss and higher moisture content, which may have enhanced the light scattering effect within the meat, leading to an increase in the L* value. This finding was consistent with Ismail et al. (2019), who noted that changes in the L* value of meat were strongly associated with the light scattering effect, which was influenced by moisture content and alterations in protein structure. Specifically, when the moisture content of meat was high, the penetration of light in muscle tissue was increased, which resulted in a rise in the L* value. In contrast, the a* value of the SV + Bro group showed no significant change compared to the SV group, indicating that bromelain had little influence on the redness value of beef during SV cooking. Meanwhile, it was also noted that the b* value in the control group was considerably greater than that of the SV + Bro group, primarily due to the production of metmyoglobin, which generates a brownish color at high temperatures (Suman et al., 2016).

### 3.7 The effect of cooking methods on the pH value

The pH value was an important indicator for assessing beef quality, a higher pH value indicated that acidic groups in the protein structure were significantly reduced after processing (Crichton et al., 2017). As illustrated in Fig. 3d, the pH measurements of beef in the SV and SV + Bro groups were 6.081 and 6.034, respectively, both significantly lower than the control group's value of 6.147 (*P* < 0.05). This was because high temperatures caused the breakdown of chemical bonds within the protein structure, which led to a decrease in acidic groups. Additionally, the introduction of bromelain resulted in the cleavage of peptide bonds in meat proteins, transforming them into smaller peptides and free amino acids. This process released carboxyl and amino groups in either free or protonated forms, ultimately resulting in a lowered pH (Gallego et al., 2023). This observation aligns with the results reported by Kadıoğlu et al. (2019), in which chicken meat was soaked in pineapple juice, revealing a lower pH value in comparison to the un-marinated state.

### 3.8 The effect of cooking methods on the MFI

**Fig. 4:**
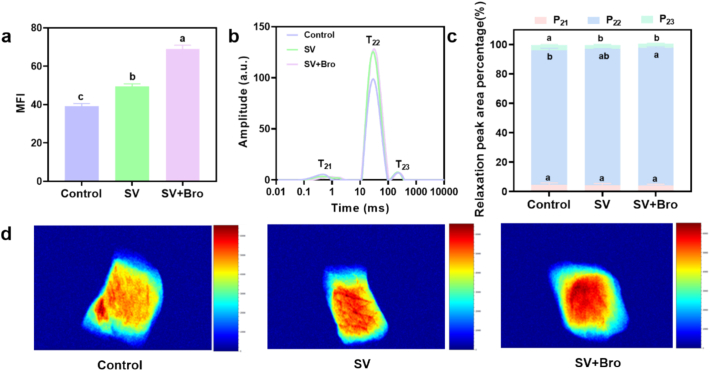
Magnetic resonance analysis of different cooking methods on the fragmentation index of beef myofibrils and moisture characteristics. (a) Effect of cooking methods on the MFI. (b) T_2_ relaxation spectra of beef under different cooking methods. (c) Peak area ratio of each moisture content in beef under different cooking methods. (d) Magnetic resonance images of beef under different cooking methods. The results were presented as mean ± standard deviation, with different letters indicating significant differences among the samples (*P* < 0.05).

MFI was typically indicative of the degree of myofibrillar degradation and protein fragmentation, where higher MFI was linked to enhanced meat tenderness and decreased shear force (Cruz et al., 2020). As depicted in Fig. 4a, the MFI was significantly elevated in both the SV and SV + Bro groups compared to the control group (*P* < 0.05), with the SV + Bro group showing the highest MFI, 1.760 times that of the control group. This increase was attributed mainly to the denaturation of myofibrillar proteins and the breakdown of collagen during SV treatment at lower temperatures and extended durations, leading to loosened and easily fractured myofibrillar structures (Li et al., 2019). Additionally, the incorporation of bromelain hydrolyzed myosin and actin, further increasing the degree of myofibrillar fragmentation. The research conducted by Naqvi et al. (2021) demonstrated that combining SV cooking with ginger protease increased MFI, consistent with the present findings.

### 3.9 The effect of cooking methods on water migration

LF-NMR was used to analyze the relaxation characteristics of hydrogen atoms in a magnetic field, allowing the measurement of the T_2_ relaxation time of moisture in samples at a microscopic level, and thereby analyzing moisture distribution (Liang et al., 2021). As shown in Fig. 4b, the three relaxation peaks represent three states of water: T_21_ for bound water, T_22_ for immobilized water, and T_23_ for free water (Wei et al., 2024). Compared to the control group, a significant increase in T_22_ was observed in both the SV and SV + Bro groups. Furthermore, as illustrated in Fig. 4c, the ratio of peak areas associated with relaxation indicated the proportion of water content in various states. In the figure, P_21_, P_22_, and P_23_ were linked to the percentage of peak areas for the relaxation times T_21_, T_22_, and T_23_, respectively. When comparing the SV and SV + Bro groups to the control group, no significant effect was observed for P_21_ (*P* > 0.05), whereas P_22_ and P_23_ displayed significant differences (*P* < 0.05). Specifically, P_22_ increased and P_23_ decreased, indicating that the distribution of immobilized water and free water in beef was mainly affected. Additionally, during conventional cooking, high temperatures destroyed the intracellular environment, causing water to flow out, loosening the cell space, thus weakening the binding force between intercellular water and converting immobilized water into free water (Li et al., 2020). Furthermore, high temperatures caused protein denaturation, leading to the separation of some water molecules bound to protein, converting them into immobilized water. Protein denaturation also damaged the integrity of muscle tissue, affecting the texture of the meat (Li et al., 2022). In the present study, texture profile analysis (Table 2) showed that the hardness, springiness, chewiness, and cohesiveness of beef subjected to SV cooking were significantly lower than those of the traditional cooking group. This trend in texture characteristics was consistent with the change pattern of immobile water.

In the MRI, stronger red values indicated a higher water content. The transition from red to yellow, green, and blue suggested a decrease in hydrogen proton density, corresponding to a lower water content (Li et al., 2022). As shown in Fig. 4d, the SV + Bro group exhibited the most red regions, suggesting the highest moisture content, aligning with the conclusions drawn about water migration.

### 3.10 Cluster analysis and PCA

#### 3.10.1 Cluster analysis

**Fig. 5:**
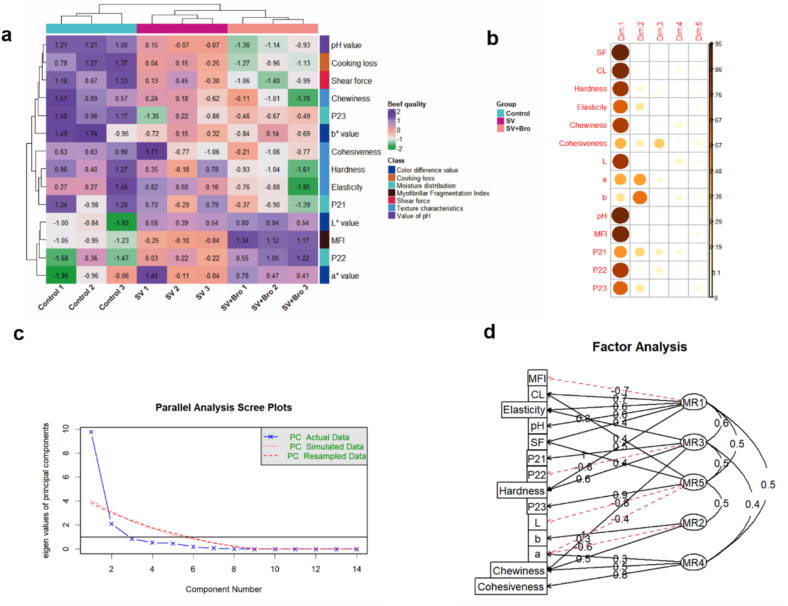
Cluster analysis and principal component analysis of the quality characteristics of beef based on cooking methods. (a) The clustering heatmap analysis of cooking methods on beef quality characteristics. (b) Correlation between various factors and principal components. (c) Principal component analysis scatter plot. (d) Cluster relationship between various factors and principal components. SF represents shear force, CL represents cooking loss.

The differences in beef quality indicators across various cooking methods are illustrated in Fig. 5a. Significant differences were observed between different indicators. From the dendrogram on the left, the fourteen indicators were mainly clustered into three categories: the first category included pH value, cooking loss, shear force, chewiness, P_23_, and b* value; the second category comprised cohesiveness, hardness, springiness, and P_21_; and the third category consisted of L* value, MFI, P_22_, and a* value. The dendrogram on the top showed that beef samples with the same treatment method exhibited high similarity in indicator characteristics. Notably, the control group exhibited higher levels of pH value, cooking loss, shear force, and hardness compared to the SV and SV + Bro groups. This suggests that cooking at elevated temperatures led to increased juice loss, thereby resulting in tougher meat. Moreover, the SV + Bro group exhibited lower shear force and hardness compared to the SV group, suggesting that the combination of SV cooking with bromelain treatment was more effective in improving meat quality. These characteristic differences provided a foundational basis for subsequent principal component analysis, facilitating a more systematic investigation into the effects of various cooking methods on beef quality.

#### 3.10.2 PCA

Fig. 5c illustrated the scree plot from the PCA. The curve leveled off at six components, indicating that six principal components could be extracted. As shown in Table 3 and Fig. S1, the cumulative variance contribution rate of PC1 and PC2 was 84.700 %, and it reached 99.427 % by PC6, suggesting that these six principal components nearly accounted for all the variability. The correlations between various factors and the main principal components, along with the cluster analysis, were shown in Fig. 5b and d. Following the minimum residual method, PC1 was found to be positively correlated with cooking loss, shear force, hardness, and elasticity (*P* < 0.01), while negatively correlated with MFI (*P* < 0.01). PC2 was positively correlated with b* value and chewiness (*P* < 0.01) and negatively correlated with a* value (*P* < 0.01). PC3 exhibited positive correlations with elasticity, P_21_, hardness, and chewiness (*P* < 0.01), and a negative relationship with P_22_ (*P* < 0.01).

**Table 3:** Initial eigenvalues, contribution rates of variance and cumulative contribution rates of variance of principal components.

PC	Initial eigenvalues	variance contribution rate (%)	cumulative variance contribution rate (%)
PC1	9.757	69.694 %	69.694 %
PC2	2.101	15.006 %	84.700 %
PC3	0.861	6.151 %	90.851 %
PC4	0.546	3.899 %	94.751 %
PC5	0.470	3.360 %	98.111 %
PC6	0.184	1.316 %	99.427 %

#### 3.10.3 Quality analysis based on mathematical models and comprehensive scores

Based on the load matrix of each indicator in Table S3, it was found that the shear force had the highest load value in PC1 (0.977), indicating that it contributed the most to PC1. In PC2, the load for b* reached its peak at 0.772, indicating that b* made the most substantial contribution to PC2. Conversely, in PC3, cohesiveness exhibited the highest load value of 0.564, illustrating that it offered the most considerable impact on PC3. In order to conduct a more thorough assessment of beef quality, the relationship equations between the first six principal components and each indicator were established using the linear combination coefficient matrix of fourteen indicators (Table S4), as follows:

*Dim*_1_ = 0.313 X_1_ + 0.302 X_2_ + 0.289 X_3_ – 0.258 X_4_ – 0.284 X_5_ – 0.202  X_6_ + 0.290  X_7_ + 0.211 X_8_ – 0.153 X_9_ – 0.308 X_10_ + 0.306 X_11_ – 0.225 X_12_ + 0.286 X_13_ – 0.259 X_14_.

*Dim*_2_ = − 0.021 X_1_ + 0.035 X_2_ – 0.198 X_3_ + 0.317 X_4_ – 0.120 X_5_ + 0.2732  X_6_ + 0.054 X_7_ + 0.472 X_8_ – 0.533 X_9_– 0.062 X_10_ – 0.046 X_11_ + 0.367 X_12_ – 0.153 X_13_ – 0.312 X_14_.

*Dim*_3_ = − 0.028 X_1_ – 0.206 X_2_ + 0.236 X_3_ + 0.105 X_4_ + 0.133 X_5_ + 0.608  X_6_ + 0.019 X_7_ + 0.324 X_8_ + 0.206 X_9_ + 0.157 X_10_ – 0.107 X_11_ – 0.409 X_12_ + 0.370  X_13_ – 0.122 X_14_.

*Dim*_4_ = 0.139 X_1_ + 0.293 X_2_ + 0.022 X_3_ – 0.049 X_4_ + 0.436 X_5_ + 0.208 X_6_ + 0.437 X_7_ – 0.088 X_8_ + 0.424 X_9_ – 0.033 X_10_ + 0.031 X_11_ + 0.370 X_12_ – 0.169 X_13_ – 0.281 X_14_.

*Dim*_5_ = − 0.019 X_1_ + 0.196 X_2_ + 0.200 X_3_ – 0.356 X_4_ + 0.272 X_5_ + 0.438 X_6_ – 0.290 X_7_ + 0.019 X_8_ – 0.262 X_9_ – 0.207 X_10_ + 0.365 X_11_ + 0.054 X_12_ – 0.209  X_13_ + 0.391 X_14_.

*Dim*_6_ = − 0.215 X_1_ – 0.011 X_2_ + 0.330 X_3_ + 0.549 X_4_ + 0.370 X_5_ – 0.286  X_6_ + 0.216 X_7_ + 0.123 X_8_ – 0.075 X_9_ – 0.321 X_10_ + 0.093 X_11_ – 0.124 X_12_ – 0.023 X_13_ + 0.281 X_14_.

The factor scores were calculated using the aforementioned formula (Table S5). Based on the variance contribution rate and cumulative variance contribution rate, a mathematical model for the comprehensive score Y under different cooking methods was established:

*Y* = 0.701 *Dim*_1_ + 0.151 *Dim*_2_ + 0.062 *Dim*_3_ + 0.039 *Dim*_4_ + 0.034 *Dim*_5_ + 0.013 *Dim*_6_.

**Fig. 6:**
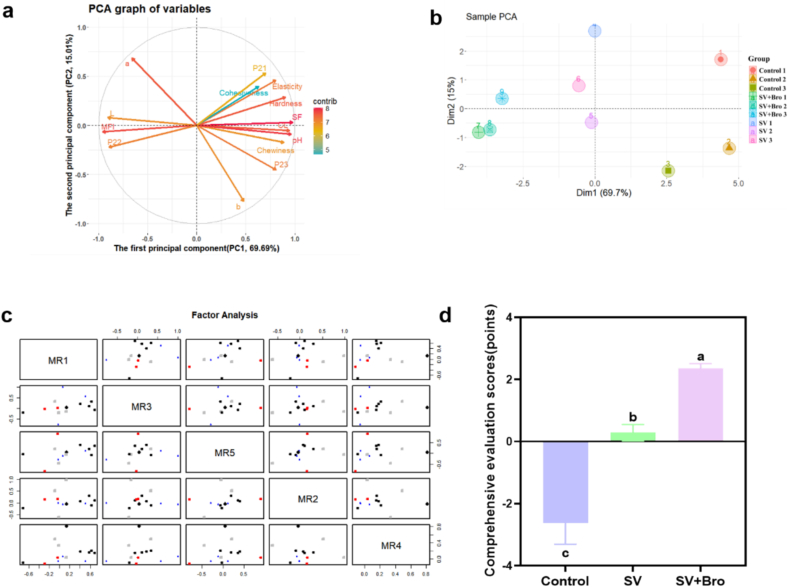
Factor analysis of quality indicators and comprehensive evaluation of different cooking methods. (a) Circle graph related to quality index factors. (b) Two-dimensional ranking graph of the principal factors in the quality indicators. (c) Factor scatter matrix diagram. (d) Comprehensive evaluation scores of different cooking methods. The results were presented as mean ± standard deviation, with different letters indicating significant differences among the samples (*P* < 0.05).

The comprehensive score, calculated using a mathematical model, is shown in Fig. 6d. Based on the principal component analysis results (Fig. 6a-c), it was determined that the quality of the beef was optimal when SV cooking was combined with bromelain treatment.

### 3.11 Histological analysis

**Fig. 7:**
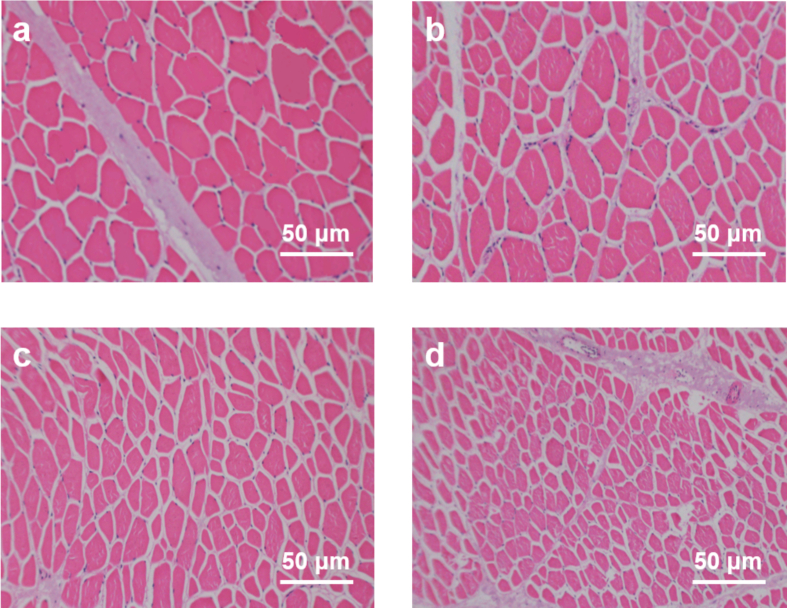
Microstructural changes in beef under different treatment conditions. (a) Untreated beef. (b) Control group. (c) SV group. (d) SV + Bro group.

Fig. 7 presented the HE-stained microstructures of beef muscle cross-sections under different treatments. In the untreated group, muscle fibers exhibited a large cross-sectional area, regular contours, and dense arrangement, with minimal inter-fiber spaces and a clearly continuous perimysium (Fig. 7a). In the control group, slight shrinkage of some fibers was observed, with the shape shifting from nearly circular to polygonal; inter-fiber gaps increased slightly, and a small number of internal voids appeared (Fig. 7b). After SV treatment, the cross-sectional area of muscle fibers was further reduced, their morphology became more irregular, and inter-fiber spaces expanded markedly. The boundaries between fiber bundles became blurred, indicating a certain degree of myofibrillar fragmentation and rearrangement (Fig. 7c). In the SV + Bro group, these changes were more pronounced: muscle fibers appeared thinner with greater size variation, and both inter-fiber spaces and local voids increased substantially, suggesting that the combined application of bromelain and SV further promoted myofibrillar protein degradation and led to a more loosened tissue structure (Fig. 7d).

It was noteworthy that due to the relatively mild enzymatic hydrolysis conditions applied in this study (30 °C, 30 min), the overall outline of the perimysium remained visible in all groups. This indicated that bromelain exhibited limited hydrolytic activity toward coarse connective tissue under such mild conditions. This observation was consistent with the findings of Xu et al. (2020), who reported that bromelain and papain mainly induced myofibrillar degradation under mild treatment conditions. Together with the significantly increased MFI and markedly reduced shear force, it could be inferred that the improvement in tenderness in this study was might attributed to two synergistic effects: (1) bromelain-induced myofibrillar fragmentation and structural loosening, and (2) thermal denaturation and partial solubilization of intramuscular collagen during SV cooking. These combined effects enhanced beef tenderness.

## 4 Conclusions

This study optimized the process conditions for tenderizing beef using a combination of SV cooking and bromelain treatment through the response surface methodology. The optimal tenderizing conditions were determined to be bromelain concentration of 250 U/mL, SV cooking time of 120 min, and SV cooking temperature of 65 °C. Additionally, the effects of different cooking methods on beef quality were analyzed. The findings showed that the SV + Bro group experienced a significant decrease in shear force, cooking loss, and hardness when compared to the control group. The L* value was significantly increased, the b* value decreased, the pH value declined, and the content of immobilized water increased, while the MFI significantly improved. PCA revealed that PC1 had a positive correlation with cooking loss, shear force, and hardness, whereas it showed a negative correlation with MFI. PC2 exhibited positive correlations with the b* value and chewiness, and negatively correlated with the a* value. PC3 was positively correlated with elasticity, P_21_, hardness, and chewiness, while negatively correlated with P_22_. The comprehensive scores indicated that the SV + Bro group achieved the highest score, suggesting the best quality. These results suggested that the combination of SV cooking technology and bromelain treatment significantly enhanced the tenderness and eating quality of beef, providing a scientifically effective method for improving beef tenderness and laying a theoretical foundation for its application in meat processing.

## CRediT authorship contribution statement

**Wei Huang:** Writing – original draft, Validation, Software, Data curation. **Lijing Geng:** Writing – review & editing, Supervision, Funding acquisition. **Hang Fu:** Validation, Data curation. **Dan Wang:** Resources. **Dongsheng Jia:** Formal analysis. **Ao Wang:** Formal analysis. **Rong Qi:** Project administration. **Mbinga Isequias Elamba Tertulliano:** Visualization. **Muhammad Zain Ul Aabideen:** Investigation. **Wei Zhou:** Writing – review & editing, Supervision. **Quimbamba Silvia Jacinta Calombe:** Methodology. **Aqsa Shafique:** Formal analysis. **Chang Liu:** Methodology.

## Declaration of competing interest

The authors declare that they have no known competing financial interests or personal relationships that could have appeared to influence the work reported in this paper.

## Data Availability

Data will be made available on request.

## Appendix A Supplementary data

Supplementary material Image 1
