# Supplementary material for: Effect of sous vide cooking combined with bromelain on beef tenderness and eating quality
Source: Food Chem X. 2025 Dec 22;33:103438. doi: 10.1016/j.fochx.2025.103438 (PMC12807838; doi:10.1016/j.fochx.2025.103438)
Supplement: Supplementary file 1 — Supplementary material [file mmc1.docx]

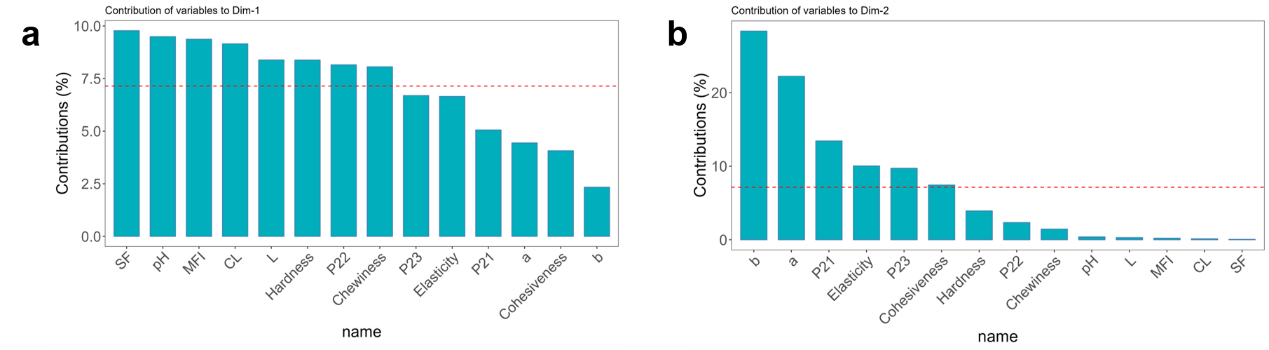


Fig. S1 Contribution of variables. (a) Contribution of variables to PC1. (b) Contribution of variables to PC2.

Table S1 Three-factors and three-levels experimental design.

| Levels |  | Factor | | |  |
| --- | --- | --- | --- | --- | --- |
|  | Bromelain concentration (U/mL) | | SV cooking time (min) | SV cooking temperature (℃) | |
| -1 | 200 | | 90 | 60 | |
| 0 | 250 | | 120 | 65 | |
| 1 | 300 | | 150 | 70 | |

Table S2 Experimental design of response surface for tenderizing beef by vacuum low-temperature cooking combined with bromelain.

| No. | A (Bromelain concentration/U/mL) | B (SV cooking time /min) | C (SV cooking temperature /℃) | Y (Shear force /N) |
| --- | --- | --- | --- | --- |
| 1 | 200 | 90 | 65 | 41.021 ± 0.328 |
| 2 | 300 | 90 | 65 | 39.533 ± 0.606 |
| 3 | 200 | 150 | 65 | 40.287 ± 1.278 |
| 4 | 300 | 150 | 65 | 39.223 ± 0.785 |
| 5 | 200 | 120 | 60 | 41.398 ± 0.978 |
| 6 | 300 | 120 | 60 | 40.627 ± 0.623 |
| 7 | 200 | 120 | 70 | 40.864 ± 1.102 |
| 8 | 300 | 120 | 70 | 38.215 ± 0.826 |
| 9 | 250 | 90 | 60 | 36.545 ± 1.974 |
| 10 | 250 | 150 | 60 | 37.265 ± 0.788 |
| 11 | 250 | 90 | 70 | 36.317 ± 0.723 |
| 12 | 250 | 150 | 70 | 35.435 ± 1.111 |
| 13 | 250 | 120 | 65 | 32.174 ± 1.218 |
| 14 | 250 | 120 | 65 | 33.591 ± 0.616 |
| 15 | 250 | 120 | 65 | 32.767 ± 1.204 |
| 16 | 250 | 120 | 65 | 32.449 ± 0.902 |
| 17 | 250 | 120 | 65 | 33.893 ± 1.207 |

Table S3 The factor loading matrices of the principal components on each index.

| Index | PC1 | PC2 | PC3 | PC4 | PC5 | PC6 |
| --- | --- | --- | --- | --- | --- | --- |
| Shear force | 0.977 | -0.031 | -0.026 | 0.103 | -0.013 | -0.092 |
| Cooking loss | 0.945 | 0.051 | -0.191 | 0.217 | 0.134 | -0.005 |
| Hardness | 0.904 | -0.287 | 0.219 | 0.016 | 0.137 | 0.142 |
| Elasticity | -0.806 | 0.459 | 0.097 | -0.036 | -0.244 | 0.255 |
| Chewiness | -0.887 | -0.174 | 0.124 | 0.322 | 0.187 | 0.159 |
| Cohesiveness | -0.630 | 0.395 | 0.564 | 0.154 | 0.300 | -0.123 |
| L* value | 0.904 | 0.078 | 0.018 | 0.350 | -0.199 | 0.093 |
| a* value | 0.658 | 0.683 | 0.301 | -0.065 | 0.013 | 0.053 |
| b* value | -0.477 | -0.772 | 0.192 | 0.313 | -0.180 | -0.032 |
| PH value | -0.962 | -0.090 | 0.146 | -0.025 | -0.142 | -0.138 |
| MFI | 0.956 | -0.067 | -0.099 | 0.023 | 0.250 | 0.040 |
| P_21_ | -0.702 | 0.531 | -0.379 | 0.274 | 0.037 | -0.053 |
| P_22_ | 0.892 | -0.221 | 0.344 | -0.125 | -0.144 | -0.010 |
| P_23_ | -0.808 | -0.452 | -0.113 | -0.207 | 0.268 | 0.121 |

Table S4 Linear combination coefficient matrix of each index.

| Variable | Index | PC1 | PC2 | PC3 | PC4 | PC5 | PC6 |
| --- | --- | --- | --- | --- | --- | --- | --- |
| X_1_ | Shear force | 0.313 | -0.021 | -0.028 | 0.139 | -0.019 | -0.215 |
| X_2_ | Cooking loss | 0.302 | 0.035 | -0.206 | 0.293 | 0.196 | -0.011 |
| X_3_ | Hardness | 0.289 | -0.198 | 0.236 | 0.022 | 0.200 | 0.330 |
| X_4_ | Elasticity | -0.258 | 0.317 | 0.105 | -0.049 | -0.356 | 0.594 |
| X_5_ | Chewiness | -0.284 | -0.120 | 0.133 | 0.436 | 0.272 | 0.370 |
| X_6_ | Cohesiveness | -0.202 | 0.273 | 0.608 | 0.208 | 0.438 | -0.286 |
| X_7_ | L* value | 0.290 | 0.054 | 0.019 | 0.473 | -0.290 | 0.216 |
| X_8_ | a* value | 0.211 | 0.472 | 0.324 | -0.088 | 0.019 | 0.123 |
| X_9_ | b* value | -0.153 | -0.533 | 0.206 | 0.424 | -0.262 | -0.075 |
| X_10_ | PH value | -0.308 | -0.062 | 0.157 | -0.033 | -0.207 | -0.321 |
| X_11_ | MFI | 0.306 | -0.046 | -0.107 | 0.031 | 0.365 | 0.093 |
| X_12_ | P_21_ | -0.225 | 0.367 | -0.409 | 0.370 | 0.054 | -0.124 |
| X_13_ | P_22_ | 0.286 | -0.153 | 0.370 | -0.169 | -0.209 | -0.023 |
| X_14_ | P_23_ | -0.259 | -0.312 | -0.122 | -0.281 | 0.391 | 0.281 |

Table S5 Each principal component and the comprehensive evaluation score.

| Group | PC1 | PC2 | PC3 | PC4 | PC5 | PC6 | Comprehensive score |
| --- | --- | --- | --- | --- | --- | --- | --- |
| Control 1 | -4.128 | 1.609 | -0.453 | -1.298 | 0.411 | 0.047 | -2.715 |
| Control 1 | -4.401 | -1.294 | -0.765 | 1.090 | 0.642 | -0.130 | -3.265 |
| Control 1 | -2.404 | -2.033 | 1.488 | -0.313 | -0.443 | -0.164 | -1.929 |
| SV 1 | 0.003 | 2.542 | 1.437 | 0.761 | -0.097 | -0.158 | 0.499 |
| SV 2 | 0.113 | -0.449 | -0.087 | -0.217 | -0.632 | 0.879 | -0.012 |
| SV 3 | 0.528 | 0.753 | -1.332 | 0.274 | -1.143 | -0.387 | 0.368 |
| SV+Bro 1 | 3.807 | -0.773 | -0.026 | -0.750 | 0.500 | -0.563 | 2.530 |
| SV+Bro 2 | 3.440 | -0.687 | -0.140 | 0.104 | -0.224 | 0.081 | 2.296 |
| SV+Bro 3 | 3.043 | 0.332 | -0.122 | 0.349 | 0.985 | 0.395 | 2.228 |
